# Endoglin involvement in integrin-mediated cell adhesion as a putative pathogenic mechanism in hereditary hemorrhagic telangiectasia type 1 (HHT1)

**DOI:** 10.3389/fgene.2014.00457

**Published:** 2015-01-07

**Authors:** Elisa Rossi, José M. Lopez-Novoa, Carmelo Bernabeu

**Affiliations:** ^1^INSERM, Faculté des Sciences Pharmaceutiques et Biologiques, Université Paris Descartes, UMR-S 1140Paris, France; ^2^Renal and Cardiovascular Research Unit, Department of Physiology and Pharmacology, University of Salamanca, and Institute of Biomedical Research of SalamancaSalamanca, Spain; ^3^Centro de Investigaciones Biológicas – Consejo Superior de Investigaciones Científicas and Centro de Investigación Biomédica en Red de Enfermedades RarasMadrid, Spain

**Keywords:** HHT, endoglin, integrins, cell adhesion, endothelial cells, inflammation, RGD

## Abstract

Mutations in the endoglin gene (*ENG*) are responsible for ∼50% of all cases with hereditary hemorrhagic telangiectasia (HHT). Because of the absence of effective treatments for HHT symptoms, studies aimed at identifying novel biological functions of endoglin which could serve as therapeutic targets of the disease are needed. Endoglin is an endothelial membrane protein, whose most studied function has been its role as an auxiliary receptor in the TGF-β receptor complex. However, several lines of evidence suggest the involvement of endoglin in TGF-β-independent functions. Endoglin displays, within its zona pellucida domain, an RGD motif, which is a prototypic sequence involved in integrin-based interactions with other proteins. Indeed, we have recently described a novel role for endothelial endoglin in leukocyte trafficking and extravasation via its interaction with leukocyte integrins. In addition, functional, as well as protein and gene expression analysis have shown that ectopic endoglin represses the synthesis of several members of the integrin family and modulates integrin-mediated cell adhesions. This review focuses on the tight link between endoglin and integrins and how the role of endothelial endoglin in integrin-dependent cell adhesion processes can provide a better understanding of the pathogenic mechanisms leading to vascular lesions in endoglin haploinsufficient HHT1 patients.

## ENDOGLIN INVOLVEMENT IN HEREDITARY HEMORRHAGIC TELANGIECTASIA

Hereditary hemorrhagic telangiectasia (HHT) or Rendu-Osler-Weber syndrome is a vascular hereditary autosomal dominant disease associated with epistaxis, telangiectasias, gastrointestinal hemorrhages, and arteriovenous malformations in lung, liver, and brain (Shovlin, 2010; McDonald et al., 2011). The first gene identified as being involved in HHT was *ENDOGLIN* (*ENG*) that maps to chromosome 9 (Fernández-Ruiz et al., 1993; McAllister et al., 1994), representing between 39 and 59% of the total HHT population, a frequency that depends on the geographic location. Several 100 different pathogenic mutations have been described in *ENG* giving rise to the HHT1 subtype (Abdalla and Letarte, 2006). Haploinsufficiency is accepted as the underlying mechanism of HHT1 pathogenicity, but the fine cellular and molecular machinery involved remains to be elucidated.

## ENDOGLIN STRUCTURE, EXPRESSION, AND FUNCTION

**FIGURE 1:**
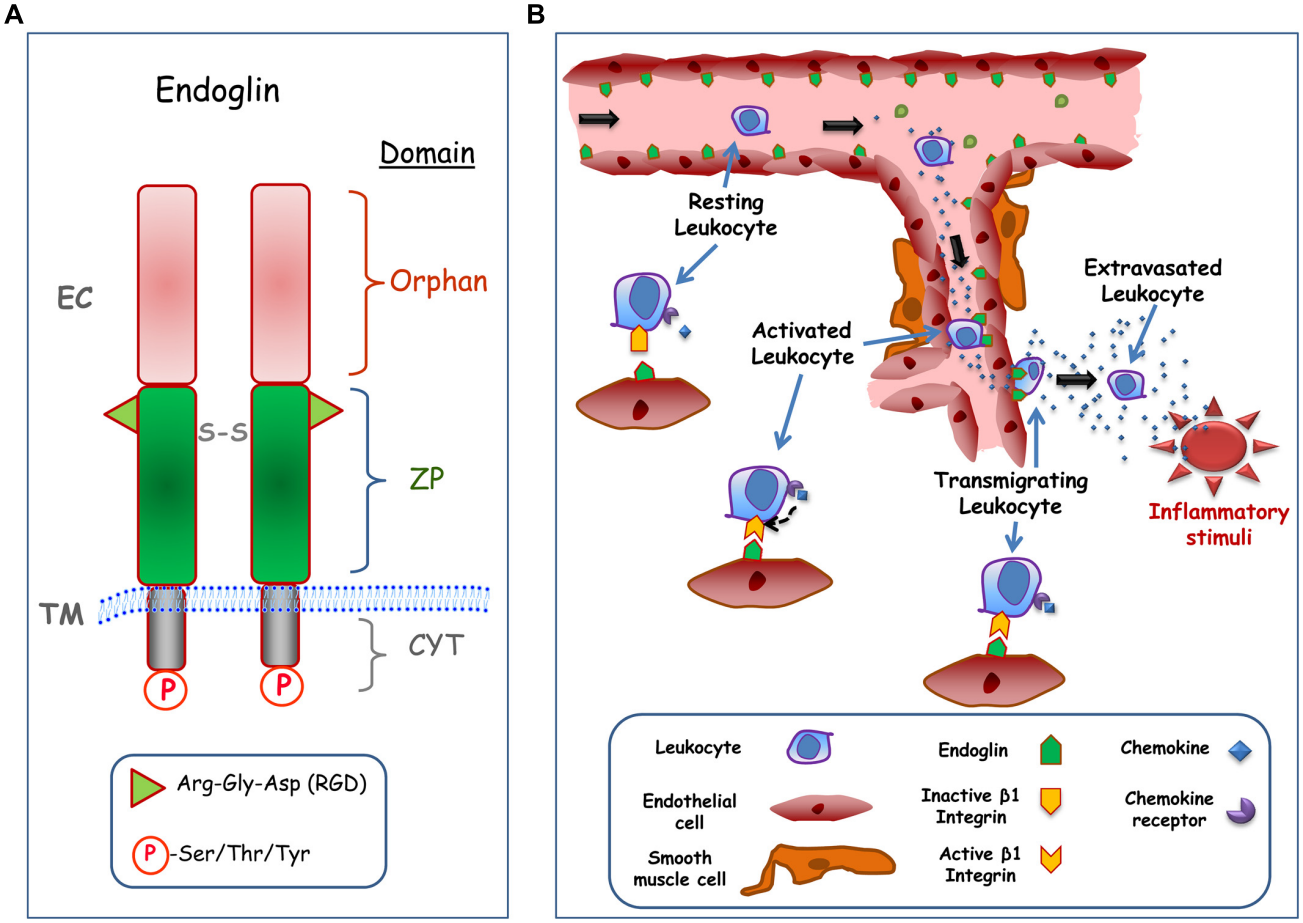
**Structure and cell adhesion function of endoglin. (A)** Structural representation of endoglin. Endoglin is a type I membrane protein with a large extracellular (EC) domain that contains a zona pellucida (ZP) domain in the juxtamembrane region and an NH_2_-terminal orphan domain. The ZP domain encodes an Arg-Gly-Asp (RGD) tripeptide that is involved in integrin binding, whereas de orphan domain is involved in binding to members of the TGF-β superfamily. Endoglin forms dimers and the corresponding monomers are disulphide linked (S-S). The cytoplasmic (CYT) domain can be phosphorylated (P) at Ser/Thr/Tyr residues. The transmembrane (TM), and EC domains of the protein are indicated. The scheme is not to scale. **(B)** Role of endothelial endoglin in leukocyte adhesion and transmigration. A schematic diagram shows a hypothetical model for leukocyte transmigration through the vessel endothelium. In an inflammatory focus, different soluble factors are released, including the chemokine CXCL12, leading to activation and endoglin-dependent extravasation of leukocytes. The transmigration process of the leukocyte involves the binding of CXCL12 to its receptor CXCR4, which in turn activates β1 integrins. Once activated, β1 integrin binds to the RGD motif of endoglin present on the endothelial cell surface, allowing the extravasation and migration of leukocytes to the inflammatory site.

Human endoglin is a type I integral membrane protein with a large extracellular (EC) domain (561 amino acids), a single hydrophobic transmembrane (TM) domain, and a short cytosolic tail (**Figure 1A**). Endoglin is a glycosylated protein expressed as a 180-kDa disulfide-linked homodimer in endothelial cells (Gougos and Letarte, 1990). The predominant expression of endoglin in endothelial cells suggests that these are the target cells in HHT, where endoglin haploinsufficiency reveals its pathogenicity. Structurally, endoglin belongs to the zona pellucida (ZP) family of proteins that share a juxtamembrane ZP domain of 260 amino acid residues in their EC region (Llorca et al., 2007). This ZP domain encodes an RGD tripeptide that is a prototypic member of a family of motifs involved in integrin-based interactions with EC matrix and certain cell surface proteins (Takada et al., 2007). Recently, it has been demonstrated that endothelial endoglin interacts with leukocyte integrin α5β1 via its RGD motif, suggesting a regulatory role for endoglin in transendothelial leukocyte trafficking (Rossi et al., 2013). The RGD motif is present in endoglin from primates, while functionally related RGD-like motifs are found in endoglin from rodents and other mammals, stressing the physiological importance and high conservation of this motif (Gougos and Letarte, 1990; Rossi et al., 2013). Next to the ZP domain is the orphan domain, which is located in the NH2-terminus of the protein (Llorca et al., 2007). The orphan domain is involved in the binding to members of the TGF-β superfamily (activin, TGF-β, and BMP families) in accordance with the role of endoglin as an auxiliary TGF-β receptor (Alt et al., 2012). Thus, endoglin modulates the endothelial response to TGF-β-related ligands, including cell proliferation, apoptosis, vascular remodeling, angiogenesis, wound healing, or fibrosis (López-Novoa and Bernabeu, 2010). Of note, most of *ENG* mutations associated with HHT1 map to the EC domain, suggesting that the biological function of this domain is critical for the disease (Bernabeu et al., 2010). The cytosolic domain of endoglin is phosphorylated at Ser/Thr/Tyr residues and it can be targeted by serine/threonine (Lastres et al., 1994) and tyrosine (Pan et al., 2014) kinases. It has been shown that the endoglin phosphorylation status can influence its subcellular localization (Koleva et al., 2006) and degradation (Pan et al., 2014). The cytoplasmic domain of endoglin also regulates actin cytoskeletal organization, microtubule-based transport machinery, and cell migration (Ray et al., 2010; Romero et al., 2010), through its specific binding to different cytosolic proteins (López-Novoa and Bernabeu, 2010).

## ENDOGLIN IN INTEGRIN-MEDIATED SIGNALING AND CELLULAR FUNCTION

Integrins are ubiquitous cell surface receptors involved in cell–cell and cell–EC matrix interactions (Takada et al., 2007) that play a relevant role in vascular biology (Plow et al., 2014). The functional role of endothelial endoglin as a counter-receptor for leukocytes’ integrins has been recently reported (Rossi et al., 2013). Interestingly, inflammatory leukocyte recruitment, which involves integrin-mediated cell–cell interactions, is critical for proper vascular remodeling. Several animal models of inflammation and vascular repair have shown that endoglin haploinsufficiency leads to an abnormal leukocyte infiltration and function that depends on the target organ or the triggering stimulus (van Laake et al., 2006; Jerkic et al., 2010; Rossi et al., 2013; Ardelean et al., 2014; Peter et al., 2014; Shen et al., 2014). Thus, an inflammation model of dextran sodium sulfate (DSS)-induced chronic colitis revealed more leukocyte infiltration in the gut and a more severe colitis phenotype in endoglin heterozygous (*Eng^+/-^*) mice relative to control animals (Jerkic et al., 2010; Ardelean et al., 2014; Peter et al., 2014). After myocardial infarction, a greater deterioration in cardiac function was observed in *Eng^+/-^* compared to control mice, although host inflammatory leukocyte numbers in the infarct zone were similar; however, defects in vessel formation and heart function in *Eng^+/-^* mice were rescued by injection of leukocytes from healthy human donors, but not by leukocytes from HHT1 patients (van Laake et al., 2006). Moreover, upon permanent distal middle cerebral artery occlusion, *Eng^+/-^* mice showed larger infarct/atrophic volumes associated with fewer infiltrating macrophages, suggesting that endoglin deficiency impairs brain injury recovery by impairing macrophage homing, delaying inflammation resolution, and reducing angiogenesis (Shen et al., 2014). Furthermore, decreased inflammation-induced leukocyte trafficking to peritoneum and lungs was found in *Eng^+/-^* mice treated with the inflammatory agents carrageenan or lipopolysaccharide (LPS), respectively (Rossi et al., 2013). The impaired leukocyte trafficking was observed for neutrophils, lymphocytes, monocytes, and basophiles. The nature of this migratory process was assessed using *in vitro* cell adhesion and transmigration studies. Leukocytes treated with the chemokine CXCL12, an integrin activator, strongly adhered to endoglin-coated plates and to endoglin-expressing endothelial cells. Similarly, leukocyte transmigration through adherent cell monolayers was dependent on the presence of endoglin. Both, endoglin-dependent cellular adhesion and transmigration processes involved the leukocyte integrin α5β1 via the endoglin RGD motif (Rossi et al., 2013). Based on these results, a hypothetical model is illustrated in **Figure 1B**.

Additional lines of evidence support a tight link between integrins and endoglin functions: (i) on overexpression of endoglin, fibroblasts tend to form clusters that involve α5β1 integrin and are inhibited by coating the culture plates with an RGD-containing fragment of fibronectin (Guerrero-Esteo et al., 1999); (ii) analysis of integrin-rich sites of focal adhesion, isolated with RGD-labeled microspheres, shows that endoglin regulates cell migration and focal adhesion composition via interaction through its cytoplasmic domain with the protein zyxin present in focal adhesion sites (Conley et al., 2004); (iii) binding of human pathogenic bacteria to the carcinoembryonic antigen (CEA) in epithelial cells, triggers *de novo* expression of endoglin, which, in turn changes focal adhesion composition, activating β1 integrins and inducing a dramatic increase in the ECM-binding capacity of the cells (Muenzner et al., 2010); (iv) endoglin mediates fibronectin/α5β1 integrin and TGF-β pathway crosstalk in endothelial cells via the internalization of the cell surface α5β1 integrin/endoglin complex (Tian et al., 2012); and (v) protein and gene expression analysis have shown that ectopic endoglin represses the synthesis of several members of the integrin family and modulates integrin-related biological functions, including cell adhesion and transmigration (Aristorena et al., 2014; Blanco et al., 2014). Moreover, endoglin is able to antagonize the integrins’ downstream MAPK pathway, by inhibiting ERK signaling and altering the subcellular distribution of activated ERK (Lee and Blobe, 2007; Santibanez et al., 2010). Taken together, these data strongly suggest a close physical and functional association between endoglin and integrins and prompt the question of whether the adhesion role of endothelial endoglin, as a counter-receptor for leukocytes’ integrins, is involved in the HHT pathogenesis.

## IS THE ROLE OF ENDOGLIN IN CELL–CELL ADHESION INVOLVED IN HHT PATHOGENESIS?

**FIGURE 2:**
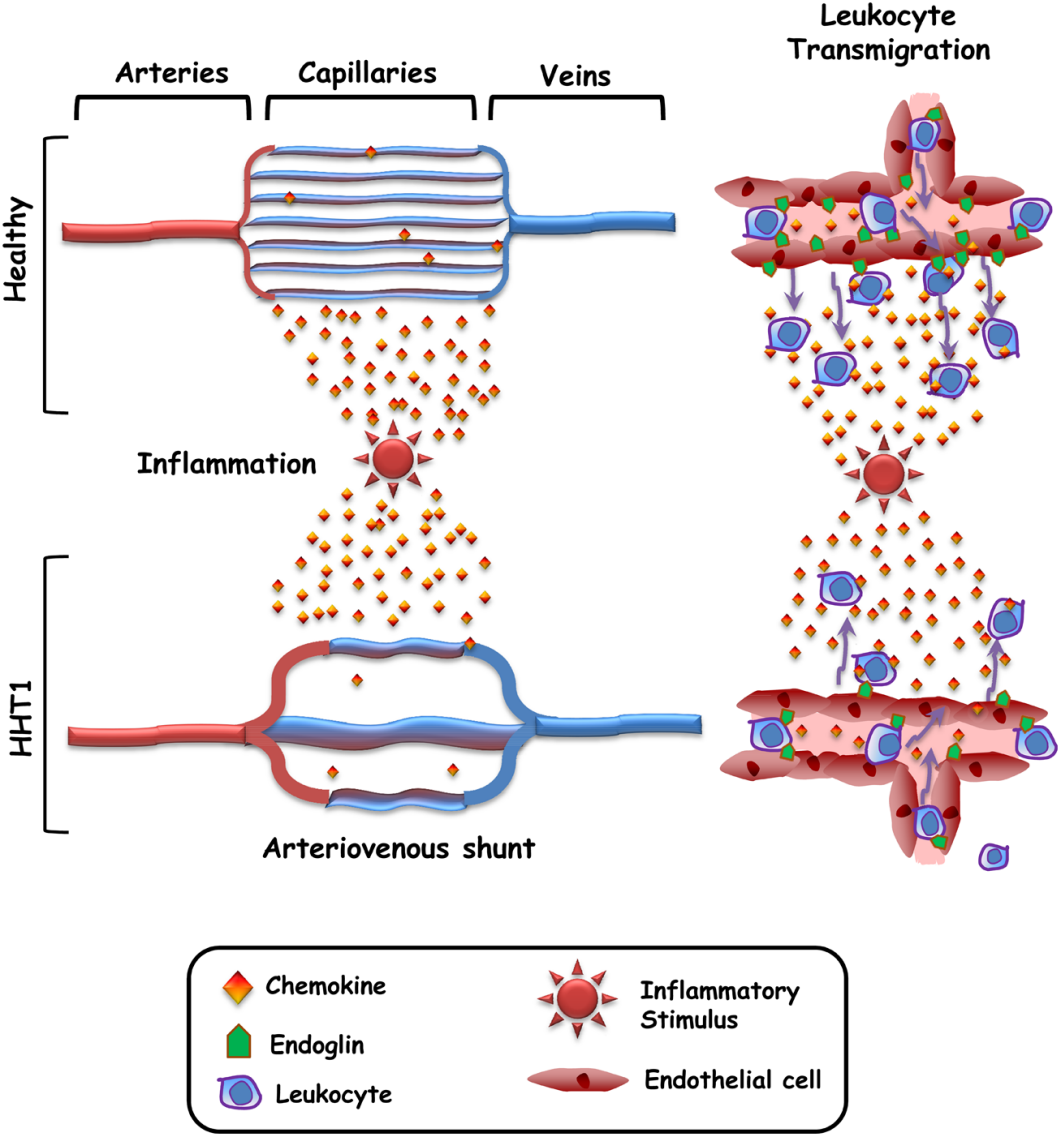
**Hypothetical model for the generation of arteriovenous malformations in HHT.** In healthy subjects, the capillary network subjected to an inflammatory stimulus is infiltrated with leukocytes that contribute to the vascular repair/remodeling. As a consequence the capillary network is restored. In HHT1 patients, endoglin haploinsufficiency impairs leukocyte adhesion/transmigration, leading to a defective vascular repair/remodeling. As a consequence, the capillary network gradually disappears and only a preferential vessel remains that eventually becomes the arteriovenous shunt.

A common feature in HHT patients is the presence of vascular lesions (telangiectasias and arteriovenous malformations) that lead to a loss of the intervening capillary network that connects the arteriole with the venule (Braverman et al., 1990). However, it remains unclear why the vascular lesions appear only at distinct sites within certain organs, rather than being present throughout the body and in all organs/tissues. To explain this paradox, the need for a trigger such as inflammation, vascular injury, angiogenic stimuli, or ischemia has been postulated (Park et al., 2009; López-Novoa and Bernabeu, 2010; Choi et al., 2014). In HHT1, this trigger would synergize with endoglin haploinsufficiency to generate the vascular lesion (Lopez-Novoa, 2012; Choi et al., 2014). Interestingly, those stimuli are usually associated with the upregulated expression of endoglin in endothelial cells and an inflammatory cell infiltrate (Torsney et al., 2002; López-Novoa and Bernabeu, 2010), suggesting the need of both, endoglin function and leukocyte infiltration, in the vascular repair/remodeling process. Indeed, it has been reported that endoglin plays a crucial role in leukocyte-mediated vascular repair (van Laake et al., 2006). Of note, HHT skin telangiectasias show a perivascular mononuclear cell infiltrate, including lymphocytes and monocytes/macrophages (Braverman et al., 1990). This is in agreement with the leukocyte infiltration occurring during angiogenesis and vascular remodeling (Jaipersad et al., 2014). Therefore, it can be hypothesized that the function of endothelial endoglin as an adhesion counter-receptor for leukocyte’s integrins is involved in HHT1 pathogenesis (**Figure 2**). According to this hypothetical model, in healthy subjects the capillary network subjected to an inflammatory stimulus is infiltrated with leukocytes that contribute to the vascular repair/remodeling. By contrast, in HHT1 patients endoglin haploinsufficiency impairs leukocyte infiltration, leading to a defective vascular repair/remodeling. As a consequence, the capillary network would disappear and only a preferential vessel remains that eventually becomes the arterio-venous shunt.

## CONCLUSION AND PERSPECTIVES

A novel role for endothelial endoglin as a cellular adhesion receptor has been reported. Here, we postulate a model attempting to relate the cell adhesion function of endoglin to the pathobiology of HHT. In the HHT1 setting, endoglin protein levels may not reach the minimum threshold to achieve the optimal function in leukocyte adhesion mediated by integrins. This endoglin haploinsufficiency alters leukocyte transmigration and may synergize with a trigger such as inflammation to generate the vascular lesion. The function of endoglin as an adhesion counter-receptor for integrins may help to understand the pathogenicity in HHT and may be a potential therapeutic target in HHT. Modulators of endoglin/integrin cell adhesion are putative drugs to be tested. In addition to the endothelial cell–leukocyte adhesion, it would be of interest to investigate whether the adhesion of endothelial cells with mural cells also involves the endoglin/integrin interaction.

## Conflict of Interest Statement

The authors declare that the research was conducted in the absence of any commercial or financial relationships that could be construed as a potential conflict of interest.
